# Association of Prediagnosis Obesity and Postdiagnosis Aspirin With Survival From Stage IV Colorectal Cancer

**DOI:** 10.1001/jamanetworkopen.2022.36357

**Published:** 2022-10-14

**Authors:** Jennifer S. Davis, Janelle C. Chavez, Melissa Kok, Yazmin San Miguel, Hwa Young Lee, Henry Henderson, Michael J. Overman, Van Morris, Bryan Kee, David Fogelman, Shailesh M. Advani, Benny Johnson, Christine Parseghian, John Paul Shen, Arvind Dasari, Kenna R. Shaw, Eduardo Vilar, Kanwal P. Raghav, Imad Shureiqi, Robert A. Wolff, Funda Meric-Bernstam, Dipen Maru, David G. Menter, Scott Kopetz, Shine Chang

**Affiliations:** 1 Department of Epidemiology, The University of Texas MD Anderson Cancer Center, Houston; 2 Now with Department of Cancer Biology, University of Kansas Medical Center, Kansas City; 3 Department of Cancer Prevention Research Training Program, The University of Texas MD Anderson Cancer Center, Houston; 4 Now with Stanford University School of Medicine, Stanford, California; 5 Now with Baylor College of Medicine, Houston, Texas; 6 Now with Abbott Laboratories, Chicago, Illinois; 7 Department of Cancer Systems Imaging, The University of Texas MD Anderson Cancer Center, Houston; 8 Now with Foundation Medicine, Atlanta, Georgia; 9 Department of Gastrointestinal Medical Oncology, The University of Texas MD Anderson Cancer Center, Houston; 10 Now with Merck & Co, Philadelphia, Pennsylvania; 11 Now with Terasaki Institute of Biomedical Innovation, Los Angeles, California; 12 Department of Sheikh Khalifa Nahyan Ben Zayed Institute for Personalized Cancer Therapy, The University of Texas MD Anderson Cancer Center, Houston; 13 Department of Clinical Cancer Prevention, The University of Texas MD Anderson Cancer Center, Houston; 14 Now with Department of Cancer Biology, University of Michigan Medical School, Ann Arbor; 15 Department of Investigational Cancer Therapeutics, The University of Texas MD Anderson Cancer Center, Houston; 16 Department of Pathology, The University of Texas MD Anderson Cancer Center, Houston

## Abstract

**Importance:**

The potential relationship between obesity and colorectal cancer (CRC) outcome is poorly understood in patients with late-stage disease. Increased body mass index may negate aspirin use for cancer prevention, but its role as a factor on the effectiveness of postdiagnosis aspirin use is unclear.

**Objective:**

To evaluate how prediagnosis obesity and postdiagnosis aspirin use may be associated with overall survival in patients with late-stage colorectal cancer.

**Design, Setting, and Participants:**

This cross-sectional study used self-reported data from patients with metastatic or treatment-refractory disease who consented to a clinical protocol at MD Anderson Cancer Center, a large US cancer treatment center. Patients were enrolled between 2010 and 2018 and followed up for mortality through July 2020. Analyses were conducted through March 2022.

**Exposures:**

Body mass index in the decade prior to initial diagnosis and regular aspirin use at survey completion.

**Main Outcomes and Measures:**

Overall survival was measured from stage IV diagnosis until death or last follow-up. Cox proportional hazards models were constructed to estimate associations of prediagnosis obesity and postdiagnosis aspirin use with overall survival.

**Results:**

Of 656 patients included in this analysis, 280 (42.7%) were women, 135 (20.6%) were diagnosed with CRC before age 45 years, 414 (63.1%) were diagnosed between ages 45 and 65 years, and 107 (16.3%) were diagnosed at 65 years or older; 105 patients (16.0%) were Black or Hispanic, and 501 (76.4%) were non-Hispanic White. Controlling for age, sex, race, stage at initial diagnosis, and weight change between prediagnosis and survey date, patients with obesity in the decade prior to CRC diagnosis had significantly higher likelihood of death (hazard ratio, 1.45; 95% CI, 1.11-1.91) compared with those with normal prediagnosis body mass index. Furthermore, only patients with normal prediagnosis body mass index experienced significant survival benefit with postdiagnosis aspirin use (hazard ratio, 0.59; 95% CI, 0.39-0.90).

**Conclusions and Relevance:**

In this cross-sectional study, our findings suggest potentially differential tumor development in the long-term physiologic host environment of obesity. Confirmation and further evaluation are needed to determine whether prediagnosis body mass index may be used to estimate the benefit from postdiagnosis aspirin use.

## Key Points

**Question:**

Do obesity and postdiagnosis aspirin use interact to affect survival in patients with stage IV colorectal cancer?

**Findings:**

In this cross-sectional analysis, obesity before colorectal cancer diagnosis was associated with significantly worse overall survival. For those who had a normal body weight before diagnosis, adjuvant aspirin use was associated with significantly improved survival.

**Meaning:**

These results suggest that obesity prior to diagnosis may influence tumor biology and outcomes despite weight change following diagnosis and, if confirmed, obesity history may be useful in projecting benefit from postdiagnosis or adjuvant aspirin use.

## Teaser

This cross-sectional study analyzes the association of prediagnosis obesity and postdiagnosis aspirin use with overall survival for US adults with stage IV colorectal cancer.

## Introduction

Colorectal cancer (CRC) is the second most common malignant neoplasm type in the US, in men and women combined. Approximately 39% of CRC patients are diagnosed with localized-stage disease, for which the 5-year relative survival rate is 90%. For those diagnosed with regional or distant disease, 5-year survival declines to 71% or 14%, respectively.^1^ Mortality is most often attributed to late stage and metastatic disease; therefore, new strategies to improve survival in this group are urgently needed.^2^

Several CRC risk factors have been established, and emerging data suggest some of these factors may also influence survival.^3^ Excess body weight is a well-established risk factor for CRC^4,5,6,7^; however, its effect on survival is less straightforward. Some studies have reported decreased survival for obese patients compared with individuals with healthy weight,^8,9,10,11,12,13,14,15,16,17^ while others have suggested increased body mass index (BMI; calculated as weight in kilograms divided by height in meters squared) improves survival.^18,19^

In contrast to obesity, regular use of aspirin has been linked to lower CRC risk,^20,21,22,23,24,25,26,27,28,29,30^ and a growing body of evidence suggests a survival benefit among patients with regular, postdiagnosis aspirin use. Several studies have reported that regular aspirin users who develop CRC have improved overall survival compared with nonusers^31,32,33,34,35,36,37,38,39,40,41,42,43^; however, these results are inconsistent, with survival benefits potentially associated with specific tumor molecular features.^39,44,45^ Due to conflicting findings and the potentially harmful adverse effects of aspirin use, including gastrointestinal ulceration and bleeding, widespread regular use is not currently recommended for cancer prevention.

Emerging evidence suggests that increased body weight may limit the efficacy of aspirin use for CRC prevention. Specifically, a recent study found that low-dose aspirin (ie, below 100 mg per day) reduced long-term CRC risk in participants weighing less than 70 kg, but not in individuals weighing 70 kg or more.^46^ However, interactions of weight status and aspirin use on survival after CRC diagnosis among patients with metastatic disease remains unknown. Studies on the role of obesity in patients with stage IV CRC are limited due to disease-related weight loss. Specifically, weight and BMI at the time of study enrollment may not reflect weight and BMI during tumorigenesis. Because we are interested in potential differences in obesity as an exposure during tumor formation, we chose to assess BMI in the decade prior to initial diagnosis, whether first diagnosed with stage IV CRC or early-stage CRC that later progressed. We chose the increment of 10 years prior to diagnosis based on the estimation of 10 to 20 years needed to develop an invasive cancer.^47^

The purpose of this study was to assess the association of regular, adjuvant aspirin use and prediagnosis obesity as combined factors with overall survival in a group of late-stage CRC patients. We performed a cross-sectional study of patients enrolled in the Assessment of Targeted Therapies Against Colorectal Cancer (ATTACC)^48^ protocol.

## Methods

### Study Population

The study population included patients treated at the University of Texas MD Anderson Cancer Center (MDACC) who participated in the ATTACC screening protocol, a clinical research study that ran from 2010 through 2018 at MDACC (ClinicalTrials.gov identifier: NCT01196130).^48^ This protocol was approved by the MDACC institutional review board, and each participant provided written informed consent. Participants were 18 years or older and had received previous treatment with systemic chemotherapy for metastatic CRC that failed. This report conforms to the Strengthening the Reporting Studies in Epidemiology (STROBE) reporting guideline for cross-sectional studies.

Participants were invited to complete a onetime, self-administered environmental survey including demographic information, smoking history, prevalence of diabetes, data on past and current aspirin and nonaspirin, nonsteroidal anti-inflammatory drug (NSAID) use and weight history. We described patients as “ever smokers” if they reported smoking at least 100 cigarettes, all others were considered never smokers. Diabetes was specified on the survey as other than during pregnancy. Survey data were combined with data from medical records, including tumor molecular characteristics (eFigure 1 in the Supplement).

Patients self-selected racial and ethnic backgrounds from a list including White, Spanish origin, Black, Asian or Pacific Islander, American Indian or Native American, and other. ATTACC participants predominantly self-identified as non-Hispanic White. To maximize power, we compared patients who did not identify as non-Hispanic White for prediagnosis BMI, finding non-Hispanic Black and Hispanic patients had different prediagnosis BMI compared with patients of other races, but not each other; therefore, we combined Black and Hispanic patients.

### Prediagnosis Obesity and Weight Change

Patients were asked to provide weight history within the past year and for specified ages: 14 to 19 years, mid 20s, mid 30s, mid 40s, mid 50s, mid 60s, and 70 years and older. To approximate weight in the decade prior to initial diagnosis, 10 years were subtracted from age at diagnosis, and the resulting decade’s weight was used to calculate BMI. Specifically, for a patient initially diagnosed at age 56 years, weight recorded in their 40s was used as prediagnosis weight. BMI was categorized as normal weight (24.9 and below), overweight (25.0 to 29.9), or obese (30.0 and above). In one sensitivity analysis, we considered a BMI of 18 or below as underweight and removed this subset from analysis, limiting the category to 18.0 to 24.9.

Weight change was calculated using prediagnosis weight and weight within the past year (from survey data or medical record review, within 6 months of study enrollment) and categorized as weight loss (ie, loss of 10% or more of prediagnosis weight), no change (less than 10% change either direction) or weight gain (gain of 10% or more of pre-diagnosis weight).^49^ Current weight was missing for 2 participants.

### Aspirin and Nonaspirin NSAID Use

Patients reported regular use of aspirin or nonaspirin NSAIDs and were asked to specify number of pills taken daily, weekly, monthly, or yearly, along with years used. We defined aspirin use as current regular (self-identified per patient) use vs no current regular use, and any use, including former, vs none. Data on nonaspirin NSAID use were missing for 17 patients.

Aspirin dose levels were defined within the survey as baby or low-dose aspirin (81 mg) and adult aspirin (325 mg). For aspirin-containing products, we assigned a dose of 325 mg. Average daily dose was calculated as the number of pills per day multiplied by dose level and summed over all types of aspirin reported. Specifically, if a patient reported 4 adult aspirin pills per week, we calculated (4 × 325) / 7 to get an average daily dose of 185.7 mg. Data on aspirin dose were missing for 15 patients reporting any aspirin use.

### CRC Characteristics

Somatic variations were measured as part of clinical care and obtained from patient medical records, where available. For this study we specifically assessed variations in *KRAS* and *PIK3CA*. *KRAS* variations occur in approximately 50% of CRC cases and can be divided into canonical (codons 12, 13, 59, 61, 117, and 146) as previously reported^50^ and noncanonical variations (ie, all others) with unknown prognostic significance. Where available, we categorized primary tumor site as right colon (including transverse), left colon (including sigmoid), and rectum (including rectosigmoid junction).

### Mortality Follow-up

Mortality follow-up, conducted as part of the ATTACC protocol, consisted of medical record review for notice of death or date last known to be alive. For patients alive at last follow-up, the most recent date of contact (in-person, by phone, email, or letter) was used. Survival time was calculated from the date of stage IV diagnosis until death or date of last follow-up, which was censored.

### Statistical Analyses

We compared patients by prediagnosis BMI category using χ^2^ tests or ANOVA, followed by post hoc Tukey test for categorical or continuous variables, respectively. Survival patterns were visualized with Kaplan-Meier curves and compared using log-rank tests. To adjust for factors known to influence survival, we constructed Cox proportional hazards^51^ models, confirming proportionality of hazards using the supremum test for proportional hazards assumption. Adjusted survival curves were generated from each model using the DIRECTADJ option. We included an interaction term between prediagnosis BMI and postdiagnosis aspirin use based on prior studies that have suggested the relationship between aspirin benefit and BMI category may not be linear.^46^ This allowed consideration of differential influence of aspirin at each level of prediagnosis BMI. Other than mediation, all statistical analyses were performed using SAS version 9.4 software (SAS Institute). Tests with a 2-sided *P* value of <.05 were considered statistically significant.

Previous studies have suggested the relationship between BMI and overall survival may be partially or fully explained by weight change. Thus, we tested whether weight change was a significant mediator on the association between prediagnosis BMI and overall survival within the structural equation modeling (SEM) framework.^52^ In SEM, relationships between variables can be decomposed into direct and indirect (ie, mediation) effects. For the current study, the mediation model was conducted using Mplus version 7.4^53^ with maximum likelihood and Monte Carlo integration methods. Testing this effect necessitated use of a continuous weight change measure. For direct comparison, we also presented Cox models with continuous weight change.

## Results

Of 656 participants, 251 (38.3%), 238 (36.3%), and 167 (25.4%) had normal, overweight, and obese prediagnosis BMI, respectively. Most (414 patients [63.1%]) were between ages 45 and 65 years at initial diagnosis, 280 (42.7%) were women, 501 (76.4%) were non-Hispanic White, 395 (60.7%) had never smoked, and 92 (14.2%) had diabetes (Table 1). While all patients had stage IV disease at study enrollment, 384 (59.0%) were initially diagnosed with stage IV CRC and the remainder were diagnosed with earlier stage disease, which progressed prior to enrollment; 49% had tumors with wild-type *KRAS*, 77% had tumors with wild-type *PIK3CA*, 44% had a tumor located on the left-side of the colon.

**Table 1.** Baseline Characteristics According to Prediagnosis BMI Category

Variable	Patients, No. (%)				*P* value
	Total	Normal (<25)	Overweight (25-30)	Obese (≥30)	
No. of patients	656	251 (38.3)	238 (36.3)	167 (25.4)	NA
Age at initial diagnosis, y					
<45	135 (20.6)	80 (31.9)	29 (12.2)	26 (15.6)	<.001
45-65	414 (63.1)	147 (58.6)	158 (66.4)	109 (65.3)	
≥65	107 (16.3)	24 (9.6)	51 (21.4)	32 (19.2)	
Sex					
Men	376 (57.3)	80 (31.9)	176 (73.9)	120 (71.9)	<.001
Women	280 (42.7)	171 (68.1)	62 (26.1)	47 (28.1)	
Race					
Black or Hispanic	105 (16.0)	34 (13.6)	40 (16.8)	31 (18.6)	.03
Non-Hispanic White	501 (76.4)	188 (74.9)	183 (76.9)	130 (77.8)	
Other^a^	50 (7.6)	29 (11.6)	15 (6.3)	6 (3.6)	
Smoking^b^					
Ever	256 (39.3)	85 (34.0)	89 (37.6)	82 (50.0)	.004
Never	395 (60.7)	165 (66.0)	148 (62.4)	82 (50.0)	
Diabetes^c^					
Yes	92 (14.2)	18 (7.2)	31 (13.2)	43 (26.1)	<.001
No	556 (85.8)	231 (92.8)	203 (86.8)	122 (73.9)	
Stage at diagnosis^d^					
I	18 (2.8)	7 (2.8)	8 (3.4)	3 (1.8)	.15
II	53 (8.1)	17 (6.8)	17 (7.2)	19 (11.5)	
III	196 (30.1)	86 (34.5)	59 (25.0)	51 (30.7)	
IV	384 (59.0)	139 (55.8)	152 (64.4)	93 (56.0)	
Total *KRAS* variations	549	206 (37.5)	199 (36.3)	144 (26.2)	
WT	272 (49.5)	104 (50.5)	93 (46.7)	75 (52.1)	.75
Canonical	270 (49.2)	100 (48.5)	102 (51.3)	68 (47.2)	
Noncanonical	7 (1.3)	2 (1.0)	4 (2.0)	1 (0.7)	
Total *PIK3CA* variations	474	187 (39.5)	170 (35.9)	117 (24.7)	
WT	364 (76.8)	143 (76.5)	124 (72.9)	97 (82.9)	.14
Mutant	110 (23.2)	44 (23.5)	46 (27.1)	20 (17.1)	
Tumor location^e^	622	237 (38.1)	224 (36.0)	161 (25.9)	
Right	215 (34.6)	75 (31.7)	83 (37.1)	57 (35.4)	.48
Left	277 (44.5)	110 (46.4)	91 (40.6)	76 (47.2)	
Rectum	130 (20.9)	52 (21.9)	50 (22.3)	28 (17.4)	

Abbreviations: BMI, body mass index; WT, wild-type variation.

^a^ Other race includes Asian or Pacific Islander, American Indian or Alaska Native, and other or not specified.

^b^ Results on smoking include 5 missing data, including 1 patient with normal, 1 with overweight, and 3 with obese BMI.

^c^ Results on diabetes include 8 missing data, including 2 patients with normal, 4 with overweight, and 2 with obese BMI.

^d^ Results on stage at diagnosis include 5 missing data, including 2 patients with normal, 2 with overweight, and 1 with obese BMI.

^e^ Data missing on 34 tumors, including for 14 patients with normal, 14 with overweight, and 6 with obese BMI.

Patients with an obese prediagnosis BMI had the poorest survival when compared with patients with an overweight or normal prediagnosis BMI (median survival: obese, 36.1 months; 95% CI, 32.5-44.3 months vs normal, 50.5 months; 95% CI, 43.6-57.0 months) (eFigure 2A in the Supplement). No significant survival difference was observed by current BMI (eFigure 2B in the Supplement).

Weight change between prediagnosis and survey completion varied significantly by prediagnosis BMI category (eTable 1 in the Supplement). Specifically, 39.2% (98 of 250 patients), 29.4% (70 of 238 patients), and 13.3% (22 of 166 patients) of patients experienced weight gain by normal, overweight, and obese prediagnosis BMI, respectively. Current regular aspirin use was reported by 19.9% (50 of 251 patients), 26.1% (62 of 238 patients), and 31.1% (52 of 167 patients) of patients with normal, overweight, and obese prediagnosis BMI, respectively. Current nonaspirin NSAID use did not vary by prediagnosis BMI.

**Figure 1.**
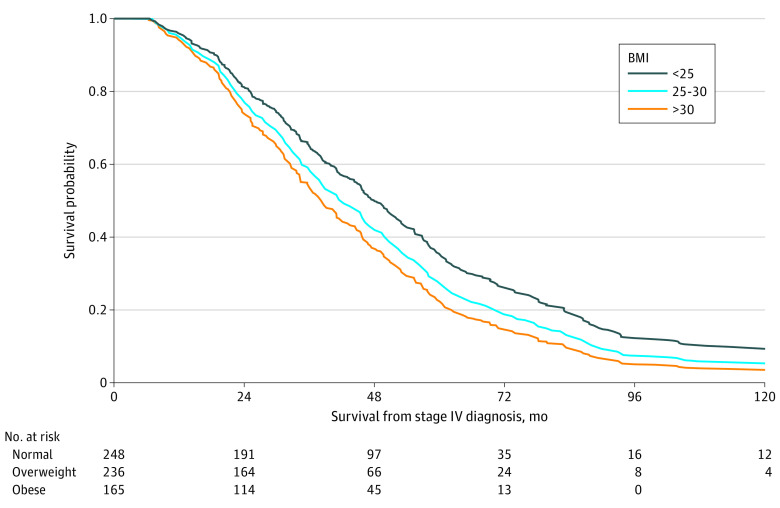
Prediagnosis BMI and Survival Estimates From Stage IV Diagnosis BMI indicates body mass index. Direct adjusted survival curves generated from the Cox proportional hazards model presented in Table 2.

Adjusting for age category, sex, race, stage at diagnosis, and weight change, patients with an obese prediagnosis BMI had increased likelihood of death compared with patients with normal prediagnosis BMI (HR, 1.45; 95% CI, 1.11-1.91) (Table 2; Figure 1). Patients with at least 10% weight gain had a lower likelihood of death compared with patients with no change (HR, 0.75; 95% CI, 0.60-0.95). Compared with patients diagnosed between ages 45 and 65 years, those diagnosed at 65 years or older had an increased likelihood of death (HR, 1.36; 95% CI, 1.05-1.74). Lastly, patients with cancer initially diagnosed at stage IV were more likely to die compared with those initially diagnosed with stage I/II disease, which then progressed (HR, 1.46; 95% CI, 1.09-1.96).

**Table 2.** Cox Proportional Hazards Model for Survival by Prediagnosis BMI

Characteristic	HR (95% CI)	*P* value
Prediagnosis BMI		
Normal weight (<25)	1 [Reference]	[Reference]
Overweight (25-30)	1.26 (0.99-1.60)	.06
Obese (≥30)	1.45 (1.11-1.91)	.007
Weight change		
No change	1 [Reference]	[Reference]
Weight loss (≥10%)	1.09 (0.84-1.41)	.53
Weight gain (≥10%)	0.75 (0.60-0.95)	.02
Age at initial diagnosis, y		
45-65	1 [Reference]	[Reference]
<45	1.18 (0.91-1.52)	.21
≥65	1.36 (1.05-1.74)	.02
Sex		
Men	1 [Reference]	[Reference]
Women	1.10 (0.90-1.35)	.35
Race and ethnicity		
Non-Hispanic White	1 [Reference]	[Reference]
Non-Hispanic Black and Hispanic	0.92 (0.71-1.19)	.53
Other^a^	0.84 (0.57-1.23)	.36
Stage at initial diagnosis		
I/II	1 [Reference]	[Reference]
III	1.26 (0.92-1.74)	.15
IV	1.46 (1.09-1.96)	.01

Abbreviations: BMI, body mass index; HR, hazard ratio.

^a^ Other race includes Asian or Pacific Islander, American Indian or Alaska Native, and other or not specified.

Patients with an overweight or obese prediagnosis BMI had an increased likelihood of death compared with patients with normal BMI before diagnosis, and this association was partially mediated by weight change (overweight: HR, 1.04; 95% CI, 1.00-1.08; obese: HR, 1.11; 95% CI, 1.02-1.21) (eTable 2, eFigure 3 in the Supplement). For direct comparison, we repeated the Cox proportional hazards model with the same variables, yielding nearly identical results (Table 2; eTable 3 in the Supplement).

Because tumor variations may be a factor for survival, we included *KRAS* and *PIK3CA* variation status in 461 patients with available data. Adjusting for factors listed in Table 2 and *KRAS* variation, patients with an obese prediagnosis BMI had increased likelihood of death compared with patients with normal prediagnosis BMI (HR, 1.48; 95% CI, 1.07-2.03) (eTable 4, eFigure 4 in the Supplement). There was also a significantly increased hazard for patients with a canonical *KRAS* variation: patients whose tumor carried a canonical *KRAS* variation had increased likelihood of death compared with those without *KRAS* variation (HR, 1.45; 95% CI, 1.16-1.82) (eTable 4, eFigure 4 in the Supplement).

**Figure 2.**
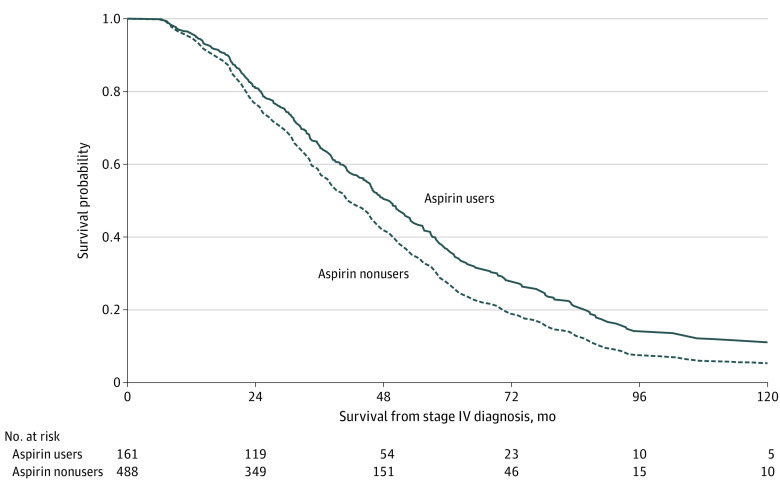
Survival Benefit Associated With Postdiagnosis Aspirin Use BMI indicates body mass index. Curves were generated from the Cox proportional hazards model shown in Table 3.

Aspirin users had a lower likelihood of death compared with nonusers (HR, 0.59; 95% CI, 0.48-0.90), with a significant interaction between prediagnosis BMI and aspirin use (Table 3). Specifically, patients with normal BMI prediagnosis experienced significant survival benefit with postdiagnosis aspirin use (HR, 0.59; 95% CI, 0.39-0.90), while patients with an overweight BMI did not; patients with an obese prediagnosis BMI experienced a nonsignificant survival benefit with postdiagnosis aspirin use (HR, 0.79; 95% CI, 0.48-1.08) (Table 3, Figure 2). Additionally, patients diagnosed at age 65 years or older were more likely to die compared with those who were ages 45 to 65 years (HR, 1.37; 95% CI, 1.07-1.77) and patients initially diagnosed with stage IV disease had a significantly higher likelihood of death compared with those diagnosed at stage I/II (HR, 1.43; 95% CI, 1.07-1.92) (Table 3).

**Table 3.** Cox Proportional Hazards Model Including Interaction Term Between Prediagnosis BMI and Postdiagnosis Aspirin Use

Characteristic	HR (95% CI)	*P* value
Prediagnosis BMI		
Normal (<25)	1 [Reference]	[Reference]
Overweight BMI (25-30)	1.11 (0.85-1.45)	.45
Obese BMI (≥30)	1.45 (1.07-1.96)	.02
Current regular aspirin use		
No aspirin use	1 [Reference]	[Reference]
Aspirin use	0.59 (0.48-0.90)	.01
**Interaction effect**		
Current regular aspirin use with prediagnosis BMI		
Aspirin use with normal BMI (<25)	1 [Reference]	[Reference]
Aspirin use with overweight BMI (25-30)	1.77 (1.03-3.07)	.04
Aspirin use with obese BMI (≥30)	1.22 (0.68-2.18)	.51
Simple main interactions^a^		
Aspirin use vs not at normal weight	0.59 (0.39-0.90)	.01
Aspirin use vs not at overweight	1.05 (0.74-1.49)	.80
Aspirin use vs not at obese	0.72 (0.48-1.08)	.11
Weight change		
No change	1 [Reference]	[Reference]
Weight loss (≥10%)	1.11 (0.85-1.44)	.45
Weight gain (≥10%)	0.79 (0.62-1.00)	.05
Age at initial diagnosis, y		
45-65	1 [Reference]	[Reference]
<45	1.14 (0.89-1.48)	.30
≥65	1.37 (1.07-1.77)	.01
Sex		
Men	1 [Reference]	[Reference]
Women	1.07 (0.87-1.32)	.50
Race and ethnicity		
Non-Hispanic White	1 [Reference]	[Reference]
Non-Hispanic Black or Hispanic	0.90 (0.69-1.17)	.43
Other^b^	0.80 (0.54-1.18)	.26
Stage at initial diagnosis		
I/II	1 [Reference]	[Reference]
III	1.25 (0.91-1.71)	.18
IV	1.43 (1.07-1.92)	.02

Abbreviation: BMI, body mass index.

^a^ The reference for each HR in this section is no aspirin use for the corresponding weight.

^b^ Other race includes Asian or Pacific Islander, American Indian or Alaska Native, and other or not specified.

To test our findings in the context of prior studies, we conducted a series of sensitivity analyses. We assessed survival by prediagnosis weight and aspirin use employing the 70 kg cutoff described in Rothwell et al.^46^ While not statistically significant, the results were in the same direction and magnitude of effect size as our initial findings (eTables 5-6, eFigures 5-6 in the Supplement). To minimize how patients with an underweight BMI may have affected our findings, we conducted a sensitivity analysis excluding patients with an underweight prediagnosis BMI (ie, below 18), showing similar results in the direction and magnitude of effect size as our original analysis (eTables 7-8, eFigures 7-8 in the Supplement). To minimize the influence of patients with very low, regular aspirin use, we reclassified patients with below 81 mg/d as aspirin nonusers, and found similar results (eTable 9, eFigure 9 in the Supplement). Lastly, to reduce the heterogeneity of stage at initial diagnosis, we restricted our population to patients initially diagnosed with stage IV disease showing no substantial difference from our original analysis (eTables 10-11, eFigures 10-11 in the Supplement).

## Discussion

Evidence for postdiagnosis aspirin use and survival from advanced stage CRC is inconsistent, and unclear regarding the role of body size. Many studies have considered BMI and its association with survival, but often these studies use only one measurement and have not evaluated the change in weight from before diagnosis.^54,55^ In this study including patients initially diagnosed with late-stage CRC and patients whose disease advanced, we found current aspirin use to be associated with better survival in patients with normal prediagnosis BMI.

Previous reports demonstrate body weight or BMI as significant factors in projecting aspirin efficacy for chemoprevention,^19,46,56^ possibly due to inadequate dosing with increased body mass. Although our study was conducted to assess aspirin use in the adjuvant setting, it was informed by previous aspirin-based prevention studies. Specifically, the Rothwell study^46^ reported CRC prevention benefit with low-dose aspirin in participants weighing less than 70 kg; participants weighing between 70 and 80 kg experienced benefit with an increased dose of 325 mg per day. However, there is inadequate evidence to support increased dosing for CRC prevention above 325 mg or in healthy populations with body weights greater than 80 kg. Rather, increasing BMI was associated with significantly increased risk of gastric ulceration independent of aspirin or nonaspirin NSAID use in the Health Professionals Follow-up Study.^57^ There is currently no consensus on how or whether to adjust aspirin dose relative to body weight, height, or BMI.^46,58^

In addition to prevention, some studies reported survival benefit with postdiagnosis aspirin use in CRC patients,^29,59^ but others found no benefit^60^ or benefit specific to patients with certain tumor molecular characteristics.^31,39,61^ Generally, such studies document a pattern of worse survival associated with increasing body size^14,37,62^ but did not simultaneously evaluate aspirin use. Similarly, these studies did not evaluate weight gain and some excluded patients with late-stage disease. In this setting, the potential for disease-related weight loss or body wasting suggests BMI near diagnosis may not reflect BMI during carcinogenesis.

A prior MDACC study^63^ found that systemic markers of inflammation were significantly elevated in metastatic CRC patients with obesity and were associated with worse outcomes, although obesity itself was not. In contrast to the previous study, we evaluated BMI in the decade prior to diagnosis as an approximation of the environment during tumorigenesis. Although we did not measure systemic inflammation, obesity-related inflammation may have led to differences in tumor biology which persist despite changes in BMI between prediagnosis and study enrollment.

Among regular aspirin users, the majority used 81 mg per day, with no significant difference by prediagnosis BMI. If systemic inflammation is significantly increased in patients with obesity, low-dose aspirin may be insufficient to overcome this; while effective for platelet inhibition,^64^ low doses of aspirin are likely ineffective to combat COX-2 driven inflammation.^65^

Interestingly, our results suggest postdiagnosis weight change partially mediated the association between prediagnosis BMI and survival. Specifically, patients with normal prediagnosis BMI were more likely to experience weight gain and had a lower likelihood of death compared with those with an overweight or obese prediagnosis BMI. Two recent clinical trial analyses found weight loss in late-stage CRC patients^66,67^ early in treatment^67^ or within 6 months prior to study enrollment^66^ independently projected poor outcomes, supporting the notion of weight loss as a potential indicator of disease progression and/or treatment toxicity. In contrast, several meta-analyses found a survival benefit among patients with postdiagnosis overweight BMI.^54,55^ Of note, the studies in these meta-analyses did not focus on late-stage CRC, which further highlights the gap in the literature filled by our study.^54^

### Strengths and Limitations

Strengths of our study included the large number of study participants included, all from a single center and with similarly advanced stage CRC, allowing our focus on survival among late-stage CRC patients. Another advantage was the availability of data from participants concerning their prediagnosis weight and current aspirin use. Observing the expected role of *KRAS* variation as well as worse survival of older patients, those diagnosed at late stage, and those who had prediagnosis obesity suggest the validity of our findings.

Our study also had several limitations. First, the inclusion of both patients initially diagnosed at stage IV and others diagnosed at earlier stages who then progressed; however, the lack of significant survival differences between these groups suggests the validity of their combination for this analysis, despite other differences. Furthermore, our inability to account for variations in treatment may lead to an overestimation of association for both prediagnosis obesity and postdiagnosis aspirin use. Limitations of a cross-sectional study apply, including lack of complete data for all participants. Importantly, weight history was self-reported and subject to recall and self-report biases. A 2019 meta-analysis^68^ found strong correlation between self-reported weight history and measured weight history, supporting use of these measures in our study. Lastly, we did not have waist circumference measures, which are a better indicator of central adiposity. Further studies are needed to delineate the influences of obesity on tumor biology and validate BMI specific associations between postdiagnosis aspirin use and survival.

## Conclusions

This study provides intriguing evidence supporting the association of obesity with CRC biology during carcinogenesis. Importantly, our findings suggest prediagnosis BMI may be useful in identifying patients who may benefit from postdiagnosis aspirin use, even in the setting of metastatic CRC.

## Notes

Supplement. **eFigure 1.** Diagram of Study Participants and Inclusion Criteria **eFigure 2.** Survival from Stage IV Diagnosis Varies Significantly by Pre-diagnosis BMI, But Not Current BMI **eFigure 3.** Weight Change Acts as a Mediator for the Relationship Between Pre-diagnosis BMI and Survival **eFigure 4.** After Adjusting for KRAS and PIK3CA Mutation, Survival From Stage IV Diagnosis Varies Significantly by Pre-diagnosis BMI **eFigure 5.** Survival From Stage IV Diagnosis by Pre-diagnosis Weight Using 70 kg Cutoff **eFigure 6.** Survival From Stage IV Diagnosis by Aspirin Use With Interaction Term Between Aspirin Use and 70 kg Weight **eFigure 7.** Survival From Stage IV Diagnosis Varies by Pre-diagnosis BMI After Removing Individuals With BMI <18 **eFigure 8.** After Removing Patients With Underweight BMI, Survival Benefit Is Observed With Aspirin Use **eFigure 9.** Survival Benefit Was Observed With Postdiagnosis Aspirin Use After Reclassifying Patients With Very Low-dose Aspirin Use as Aspirin Non-users **eFigure 10.** Survival by Pre-diagnosis BMI in Patients Initially Diagnosed With Stage IV Disease **eFigure 11.** Survival Benefit Was Observed With Post-diagnosis Aspirin Use in Patients Initially Diagnosed With Stage IV Disease **eTable 1.** Weight Change and NSAID Dose by Pre-diagnosis BMI Category **eTable 2.** Results of Mediation Analysis for Weight Change as a Mediator Between Pre-diagnosis BMI and Survival **eTable 3.** Cox Proportional Hazards Model for Survival by Pre-diagnosis BMI Including a Continuous Weight Change Variable **eTable 4.** Cox Proportional Hazards Model for Survival by Pre-diagnosis BMI Including Select Tumor Mutations, Where Available **eTable 5.** Cox Proportional Hazards Model by Pre-diagnosis Weight Using a 70 kg Cutoff for Comparison With Published Results From Prevention Trials **eTable 6.** Cox Proportional Hazards Model by Pre-diagnosis Weight Using a 70 kg Cutoff and Post-diagnosis Aspirin Use, Including an Interaction Term **eTable 7.** Cox Proportional Hazards Model by Pre-diagnosis BMI, Excluding Underweight (<18 kg/m^2^) **eTable 8.** Cox Proportional Hazards Model by Pre-diagnosis BMI and Post-diagnosis Aspirin Use, Excluding Underweight (<18 kg/m^2^) **eTable 9.** Cox Proportional Hazards Model by Pre-diagnosis BMI and Post-diagnosis Aspirin Use, Reclassifying Users With <81 mg/day as Non-users **eTable 10.** Cox Proportional Hazards Model by Pre-diagnosis BMI, Restricted to Patients Initially Diagnosed With Stage IV Disease **eTable 11.** Cox Proportional Hazards Model by Pre-diagnosis BMI and Post-diagnosis Aspirin Use, Restricted to Patients Initially Diagnosed With Stage IV Disease
