# Supplementary material for: Association of Prediagnosis Obesity and Postdiagnosis Aspirin With Survival From Stage IV Colorectal Cancer
Source: JAMA Netw Open. 2022 Oct 14;5(10):e2236357. doi: 10.1001/jamanetworkopen.2022.36357 (PMC9568800; doi:10.1001/jamanetworkopen.2022.36357)
Supplement: Supplement. — eFigure 1. Diagram of Study Participants and Inclusion Criteria eFigure 2. Survival from Stage IV Diagnosis Varies Significantly by Pre-diagnosis BMI, But Not Current BMI eFigure 3. Weight Change Acts as a Mediator for the Relationship Between Pre-diagnosis BMI and Survival eFigure 4. After Adjusting for KRAS and PIK3CA Mutation, Survival From Stage IV Diagnosis Varies Significantly by Pre-diagnosis BMI eFigure 5. Survival From Stage IV Diagnosis by Pre-diagnosis Weight Using 70 kg Cutoff eFigure 6. Survival From Stage IV Diagnosis by Aspirin Use With Interaction Term Between Aspirin Use and 70 kg Weight eFigure 7. Survival From Stage IV Diagnosis Varies by Pre-diagnosis BMI After Removing Individuals With BMI <18 eFigure 8. After Removing Patients With Underweight BMI, Survival Benefit Is Observed With Aspirin Use eFigure 9. Survival Benefit Was Observed With Postdiagnosis Aspirin Use After Reclassifying Patients With Very Low-dose Aspirin Use as Aspirin Non-users eFigure 10. Survival by Pre-diagnosis BMI in Patients Initially Diagnosed With Stage IV Disease eFigure 11. Survival Benefit Was Observed With Post-diagnosis Aspirin Use in Patients Initially Diagnosed With Stage IV Disease eTable 1. Weight Change and NSAID Dose by Pre-diagnosis BMI Category eTable 2. Results of Mediation Analysis for Weight Change as a Mediator Between Pre-diagnosis BMI and Survival eTable 3. Cox Proportional Hazards Model for Survival by Pre-diagnosis BMI Including a Continuous Weight Change Variable eTable 4. Cox Proportional Hazards Model for Survival by Pre-diagnosis BMI Including Select Tumor Mutations, Where Available eTable 5. Cox Proportional Hazards Model by Pre-diagnosis Weight Using a 70 kg Cutoff for Comparison With Published Results From Prevention Trials eTable 6. Cox Proportional Hazards Model by Pre-diagnosis Weight Using a 70 kg Cutoff and Post-diagnosis Aspirin Use, Including an Interaction Term eTable 7. Cox Proportional Hazards Model by Pre-diagnosis BMI, Excluding Un [file jamanetwopen-e2236357-s001.pdf]

Supplemental Online Content

Davis JS, Chavez JC, Kok M, et al. Association of prediagnosis obesity and postdiagnosis aspirin with survival from stage IV colorectal cancer. *JAMA Netw Open.* 2022;5(10):e2236357. doi:10.1001/jamanetworkopen.2022.36357

eFigure 1. Diagram of Study Participants and Inclusion Criteria

eFigure 2. Survival from Stage IV Diagnosis Varies Significantly by Pre-diagnosis BMI, But Not Current BMI

eFigure 3. Weight Change Acts as a Mediator for the Relationship Between Pre-diagnosis BMI and Survival

eFigure 4. After Adjusting for KRAS and PIK3CA Mutation, Survival From Stage IV Diagnosis Varies Significantly by Pre-diagnosis BMI

eFigure 5. Survival From Stage IV Diagnosis by Pre-diagnosis Weight Using 70 kg Cutoff

eFigure 6. Survival From Stage IV Diagnosis by Aspirin Use With Interaction Term Between Aspirin Use and 70 kg Weight

eFigure 7. Survival From Stage IV Diagnosis Varies by Pre-diagnosis BMI After Removing Individuals With BMI <18

eFigure 8. After Removing Patients With Underweight BMI, Survival Benefit Is Observed With Aspirin Use

eFigure 9. Survival Benefit Was Observed With Postdiagnosis Aspirin Use After Reclassifying Patients With Very Low-dose Aspirin Use as Aspirin Non-users

eFigure 10. Survival by Pre-diagnosis BMI in Patients Initially Diagnosed With Stage IV Disease

eFigure 11. Survival Benefit Was Observed With Post-diagnosis Aspirin Use in Patients Initially Diagnosed With Stage IV Disease

eTable 1. Weight Change and NSAID Dose by Pre-diagnosis BMI Category

eTable 2. Results of Mediation Analysis for Weight Change as a Mediator Between Pre-diagnosis BMI and Survival

eTable 3. Cox Proportional Hazards Model for Survival by Pre-diagnosis BMI Including a Continuous Weight Change Variable

© 2022 Davis JS et al. *JAMA Network Open.*

eTable 4. Cox Proportional Hazards Model for Survival by Pre-diagnosis BMI Including Select Tumor Mutations, Where Available

eTable 5. Cox Proportional Hazards Model by Pre-diagnosis Weight Using a 70 kg Cutoff for Comparison With Published Results From Prevention Trials

eTable 6. Cox Proportional Hazards Model by Pre-diagnosis Weight Using a 70 kg Cutoff and Post-diagnosis Aspirin Use, Including an Interaction Term

eTable 7. Cox Proportional Hazards Model by Pre-diagnosis BMI, Excluding Underweight (<18 kg/m^2)

eTable 8. Cox Proportional Hazards Model by Pre-diagnosis BMI and Post-diagnosis Aspirin Use, Excluding Underweight (<18 kg/m^2)

eTable 9. Cox Proportional Hazards Model by Pre-diagnosis BMI and Post-diagnosis Aspirin Use, Reclassifying Users With <81 mg/day as Non-users

eTable 10. Cox Proportional Hazards Model by Pre-diagnosis BMI, Restricted to Patients Initially Diagnosed With Stage IV Disease

eTable 11. Cox Proportional Hazards Model by Pre-diagnosis BMI and Post-diagnosis Aspirin Use, Restricted to Patients Initially Diagnosed With Stage IV Disease

This supplemental material has been provided by the authors to give readers additional information about their work.

© 2022 Davis JS et al. *JAMA Network Open.*

eFigure 1. Diagram of Study Participants and Inclusion Criteria

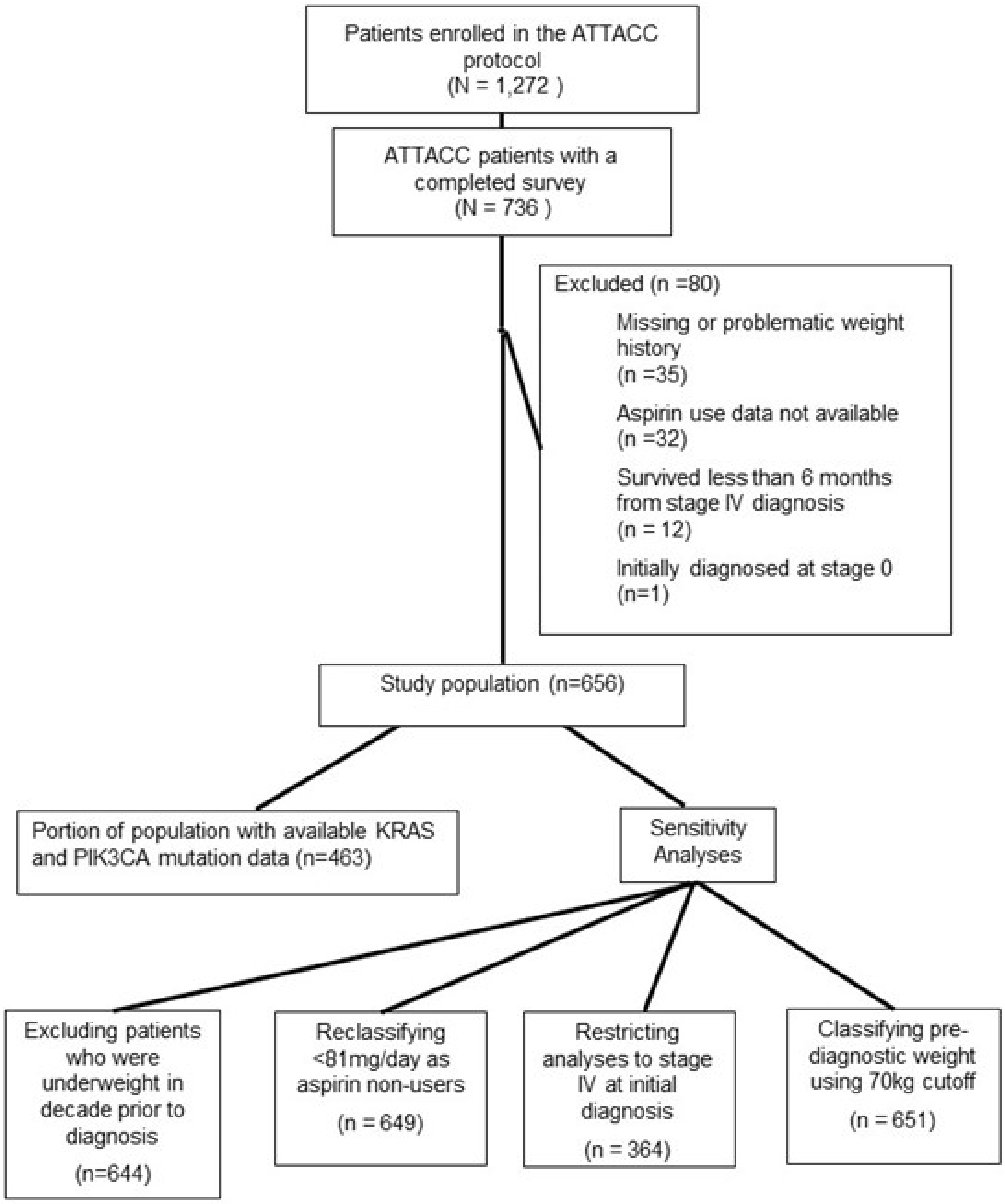

Diagram of study participants and inclusion criteria. A total of 1272 patients enrolled on the ATTACC protocol and 736 completed the survey (57.9%). Out of 736 participants

© 2022 Davis JS et al. *JAMA Network Open.*

with a completed survey, 80 (10.9%) were excluded due to missing or problematic weight history, missing aspirin use data, death within 6 months of study entrance or initial diagnosis of stage 0 disease. Of the included study population, 463 (70.6%) had available data on tumor mutations in *KRAS* and *PIK3CA*. Sensitivity analyses were conducted on four subsets of patients: excluding patients who were underweight in the decade prior to diagnosis, reclassifying patients with very low doses of aspirin as non-users, restricting analyses to patients initially diagnosed with stage IV disease and dichotomizing pre-diagnosis weight using a 70kg cutoff.

© 2022 Davis JS et al. *JAMA Network Open.*

eFigure 2. Survival from Stage IV Diagnosis Varies Significantly by Pre-diagnosis BMI, But Not Current BMI

A

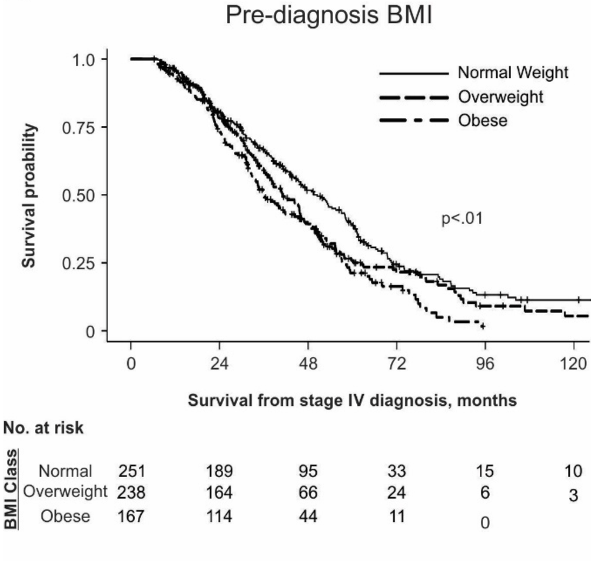

B

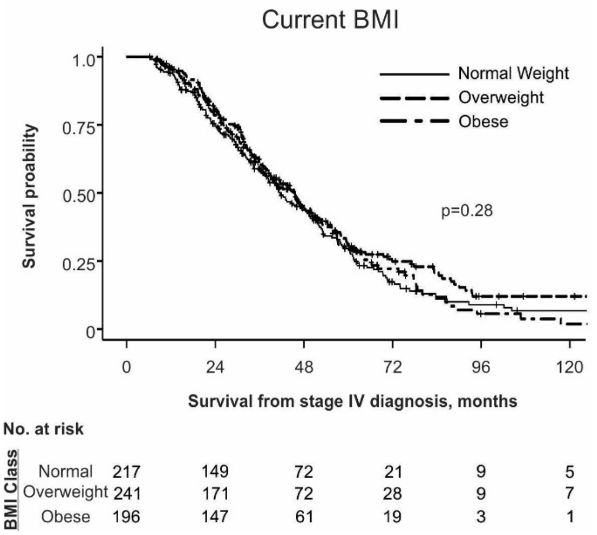

Survival from stage IV diagnosis varies significantly by pre-diagnosis BMI, but not current BMI. Kaplan Meier Curve showing survival by normal weight (solid line), overweight (dashed line) or obese (dash dot dash line) pre-diagnosis (A) or current (B) BMI. Number at risk is shown beneath the plot, aligned to the corresponding timepoint.

© 2022 Davis JS et al. *JAMA Network Open.*

eFigure 3. Weight Change Acts as a Mediator for the Relationship Between Pre-diagnosis BMI and Survival

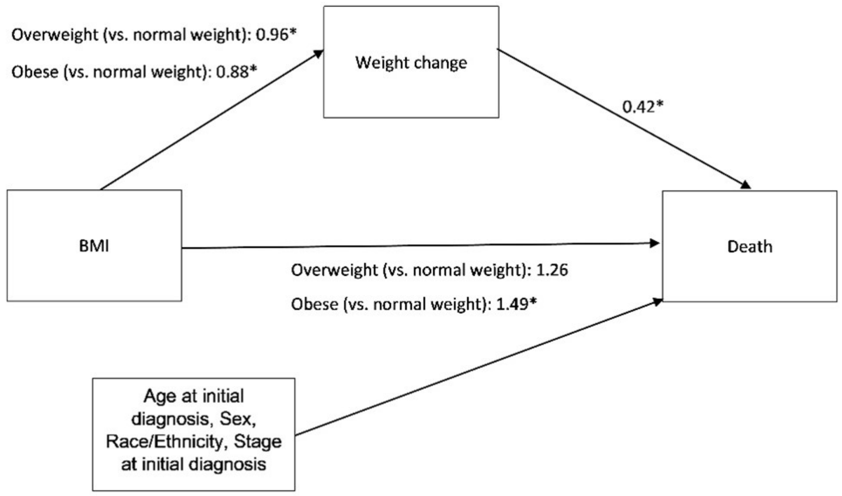

Weight change acts as a mediator for the relationship between pre-diagnosis BMI and survival. Hazard Ratios are presented for direct and indirect effects. *Indicates statistically significant result. Hazard Ratios for co-variates are shown in eTable 2.

© 2022 Davis JS et al. *JAMA Network Open.*

eFigure 4. After Adjusting for KRAS and PIK3CA Mutation, Survival From Stage IV Diagnosis Varies Significantly by Pre-diagnosis BMI

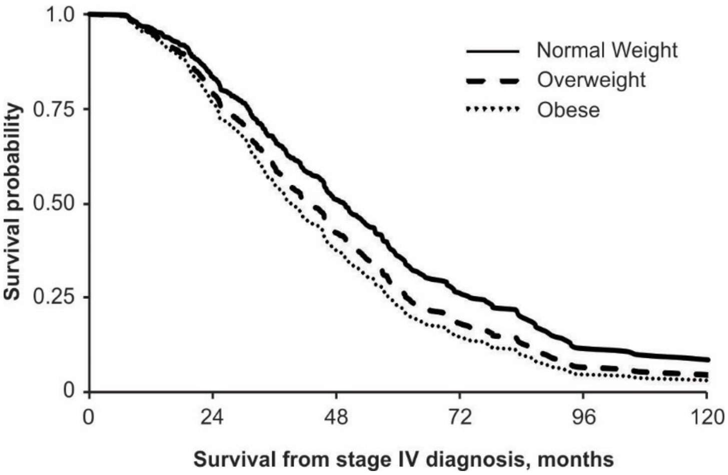

After adjusting for KRAS and PIK3CA mutation, survival from stage IV diagnosis varies significantly by pre-diagnosis BMI. Direct Adjusted survival curves generated from the Cox Proportional Hazards model shown in eTable 4. Pre-diagnosis normal (solid line), overweight (dashed line) or obese (dotted line) are shown.

© 2022 Davis JS et al. *JAMA Network Open.*

eFigure 5. Survival From Stage IV Diagnosis by Pre-diagnosis Weight Using 70 kg Cutoff

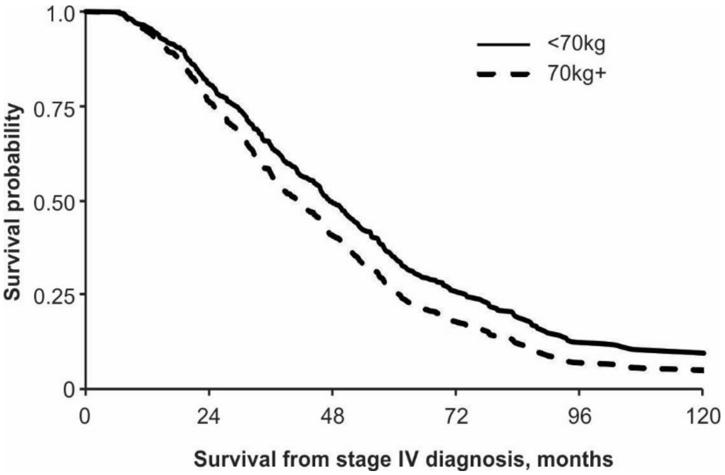

Survival from stage IV diagnosis by pre-diagnosis weight using 70kg cutoff. Direct Adjusted survival curves generated from the Cox Proportional Hazards model shown in eTable 5. Pre-diagnosis weight <70kg (solid line) and 70kg+ (dashed line) are shown.

© 2022 Davis JS et al. *JAMA Network Open.*

eFigure 6. Survival From Stage IV Diagnosis by Aspirin Use With Interaction Term Between Aspirin Use and 70 kg Weight

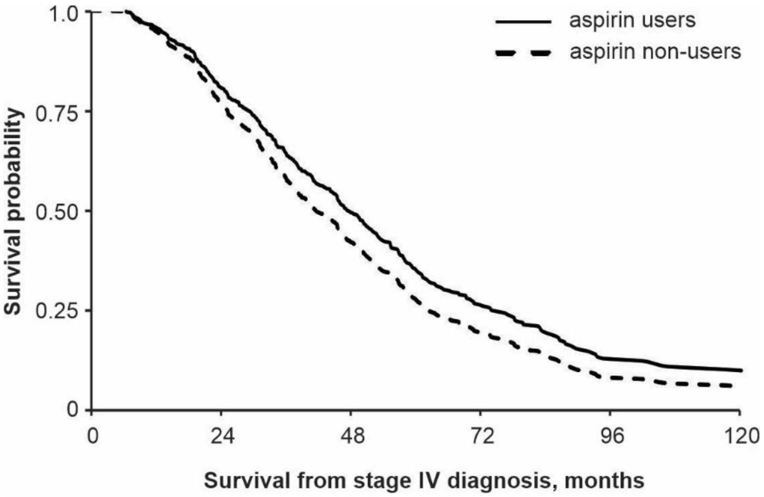

Survival from stage IV diagnosis by aspirin use with interaction term between aspirin use and 70kg weight. Direct Adjusted survival curves are shown among aspirin users (solid line) and non-users (dashed line). Curves were generated from the Cox Proportional Hazards model shown in eTable 6.

© 2022 Davis JS et al. *JAMA Network Open.*

eFigure 7. Survival From Stage IV Diagnosis Varies by Pre-diagnosis BMI After Removing Individuals With BMI <18

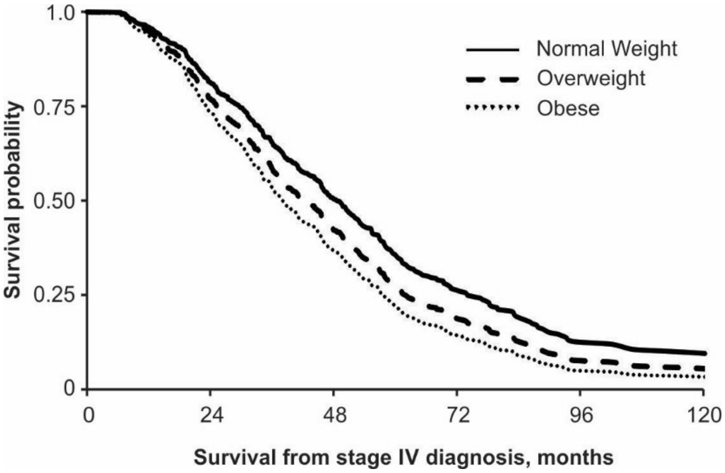

Survival from stage IV diagnosis varies by pre-diagnosis BMI after removing individuals with BMI <18. Pre-diagnosis BMI predicts survival from stage IV diagnosis after removing patients with underweight BMI. Direct adjusted survival curves generated from the Cox Proportional Hazards model shown in eTable 7.

© 2022 Davis JS et al. *JAMA Network Open.*

eFigure 8. After Removing Patients With Underweight BMI, Survival Benefit Is Observed With Aspirin Use

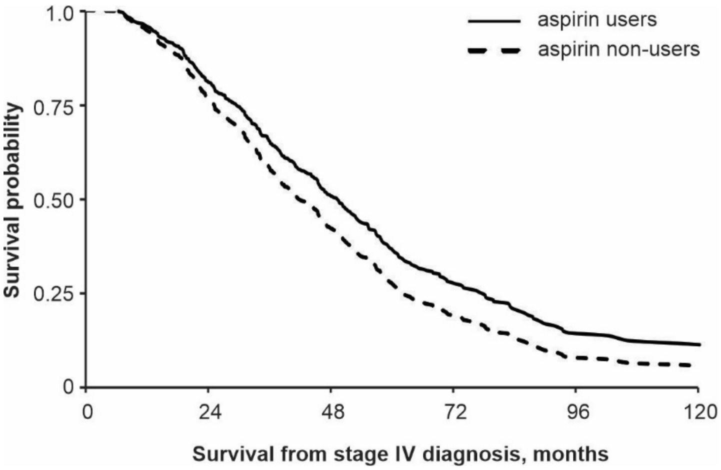

After removing patients with underweight BMI, survival benefit is observed with aspirin use. Survival is compared between aspirin users (solid line) and aspirin non-users (dashed line). Curves were generated from the Cox Proportional Hazards model shown in eTable 8.

© 2022 Davis JS et al. *JAMA Network Open.*

eFigure 9. Survival Benefit Was Observed With Postdiagnosis Aspirin Use After Reclassifying Patients With Very Low-dose Aspirin Use as Aspirin Non-users

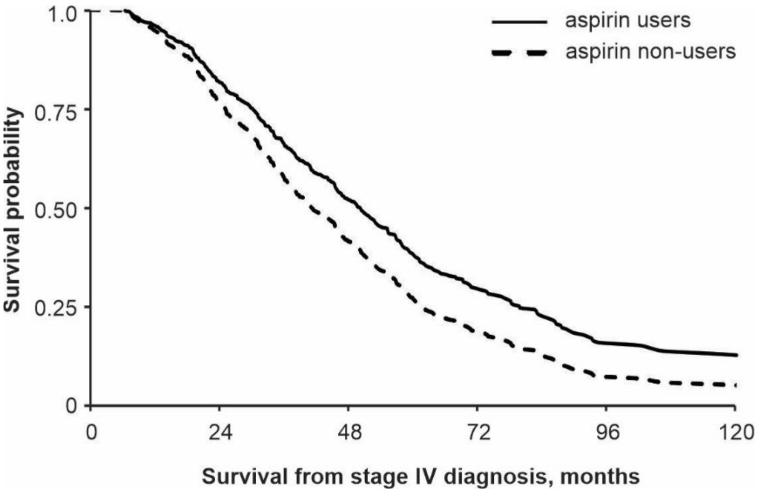

Survival benefit was observed with post-diagnosis aspirin use after reclassifying patients with very low-dose aspirin use as aspirin non-users. Direct Adjusted survival curves generated from the Cox Proportional Hazards model shown in eTable 9. Survival compared between aspirin users (solid line) and aspirin non-users (dashed line).

© 2022 Davis JS et al. *JAMA Network Open.*

eFigure 10. Survival by Pre-diagnosis BMI in Patients Initially Diagnosed With Stage IV Disease

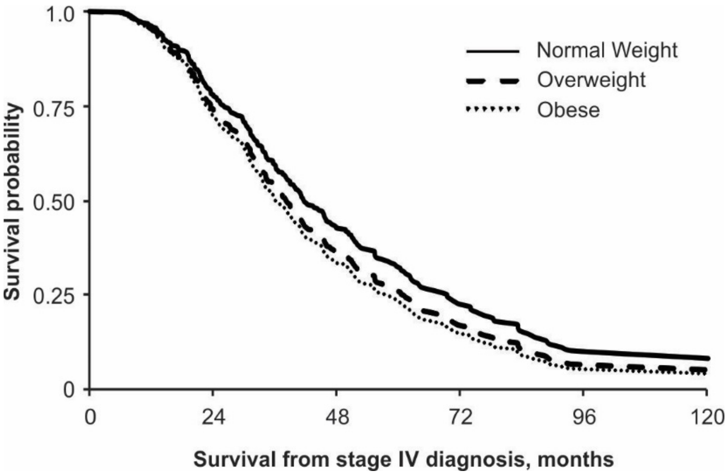

Survival by pre-diagnosis BMI in patients initially diagnosed with stage IV disease. Direct Adjusted survival curves generated from the Cox Proportional Hazards model shown in eTable 10. Pre-diagnosis normal (solid line), overweight (dashed line) or obese (dotted line) are shown.

© 2022 Davis JS et al. *JAMA Network Open.*

eFigure 11. Survival Benefit Was Observed With Post-diagnosis Aspirin Use in Patients Initially Diagnosed With Stage IV Disease

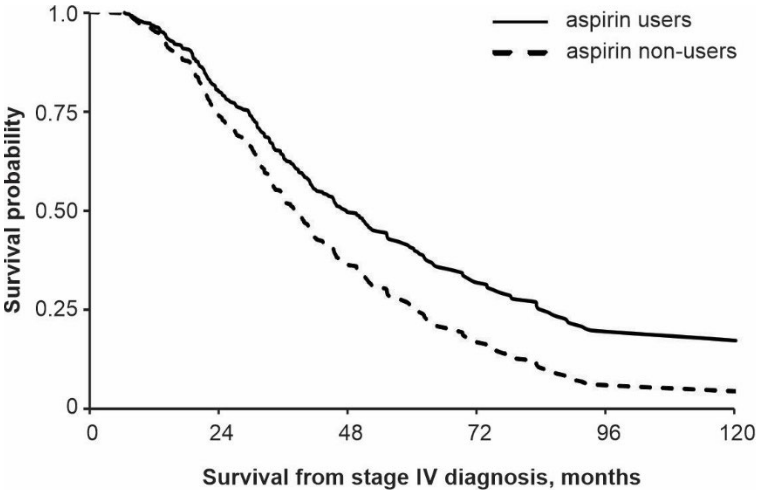

Survival benefit was observed with post-diagnosis aspirin use in patients initially diagnosed with stage IV disease. Direct Adjusted survival curves generated from the Cox Proportional Hazards model shown in eTable 11. Survival compared between aspirin users (solid line) and aspirin non-users (dashed line).

© 2022 Davis JS et al. *JAMA Network Open.*

eTable 1. Weight Change and NSAID Dose by Pre-diagnosis BMI Category

eTable 1. Weight change and NSAID use by pre-diagnosis BMI	Total	Normal Weight (BMI <25)	Overweight (BMI 25-30)	Obese (BMI 30+)	p value
Weight change[a]	654	250 (38.2)	238 (36.4)	166 (25.4)	<.001
Loss of at least 10%	114 (17.4)	16 (6.4)	38 (16.0)	60 (36.1)	
Within 10% either direction	350 (53.5)	136 (54.4)	130 (54.6)	84 (50.6)	
Gain of at least 10%	190 (29.1)	98 (39.2)	70 (29.4)	22 (13.3)	
NSAID specifics (% total)					
	656				
current aspirin	164 (25.0)	50 (19.9)	62 (26.1)	52 (31.1)	0.03
any aspirin use	188 (28.7)	61 (24.3)	71 (29.8)	56 (33.5)	0.11
	639 [b]				
any non-aspirin	277 (43.4)	109 (44.3)	96 (41.6)	72 (44.4)	0.79
current non-aspirin	260 (40.7)	104 (42.3)	89 (38.5)	67 (41.4)	0.69
Aspirin dose level, n (% of users – any use)[c]	173	54 (31.2)	66 (38.2)	53 (30.6)	0.64
<=81mg/day	120 (69.4)	40 (74.1)	45 (68.2)	35 (66.0)	
> 81mg/day	53 (30.6)	14 (25.9)	21 (31.8)	18 (34.0)	

[a]2 patients had missing data for current weight

[b]17 patients had missing data for non-aspirin NSAID use

[c]15 patients reported ‘any use’ of aspirin, but did not provide dose level or any additional information resulting in fewer case available for dose-level analysis

© 2022 Davis JS et al. *JAMA Network Open.*

eTable 2. Results of Mediation Analysis for Weight Change as a Mediator Between Pre-diagnosis BMI and Survival

eTable 2 Weight change acts as a mediator		
	HR	95% CI
Direct effect of pre-diagnose BMI on a mediator, weight change (ref=normal weight)		
Overweight BMI → Weight change	0.96	0.93, 0.98
Obese BMI → Weight change	0.88	0.86, 0.91
Direct effect of weight change on death	0.42	0.22, 0.81
Direct effect of pre-diagnose BMI on death		
Overweight BMI → death	1.26	0.99, 1.61
Obese BMI → death	1.49	1.15, 1.93
Indirect effect of pre-diagnosis BMI on death through weight change		
Overweight → weight change→ death	1.04	1.00, 1.08
Obese → weight change→ death	1.11	1.02, 1.21
Direct effect of age at initial diagnosis on death (ref=45-65)		
<45 → death	1.13	0.87, 1.46
65+ → death	1.37	1.07, 1.74
Direct effect of sex on death (ref=male)		
Female → death	1.08	0.89, 1.32
Direct effect of race on death (ref= NH Caucasian)		
NH African American and Hispanic → death	0.91	0.71, 1.18
Other → death	0.81	0.53, 1.24
Direct effect of stage at initial diagnosis on death (ref= I/II)		
Stage III	1.27	0.96, 1.68
Stage IV	1.44	1.11, 1.86
NH – Non-Hispanic, Other Race includes Asian/Pacific Islander, American Indian/Alaska Native, and Other, not specified		

© 2022 Davis JS et al. *JAMA Network Open.*

eTable 3. Cox Proportional Hazards Model for Survival by Pre-diagnosis BMI Including

eTable 3, Cox proportional Hazards model for survival by pre-diagnosis BMI with continuous weight change variable		
N=649	HR (95% CI)	p value
Pre-Diagnosis BMI		
Normal Weight (<25) (Ref)	1.00	
Overweight (25-30)	1.26 (0.99, 1.60)	0.06
Obese (30+)	1.44 (1.10, 1.88)	0.01
Percent Weight Change (continuous)	0.99 (.099, 1.00)	<.01
Age at initial diagnosis		
<45	1.15 (0.90, 1.48)	0.26
45-65 (Ref)	1.00	
65+	1.36 (1.06, 1.75)	0.02
Sex		
Male (Ref)	1.00	
Female	1.10 (0.90, 1.34)	0.36
Race/Ethnicity		
NH African American and Hispanic	0.93 (0.72, 1.21)	0.60
NH Caucasian (Ref)	1.00	
Other	0.84 (0.57, 1.23)	0.36
Stage at initial diagnosis		
I/II (Ref)	1.00	
III	1.25 (0.91, 1.72)	0.26
IV	1.45 (1.08, 1.94)	0.01
NH – Non-Hispanic, Other Race includes Asian/Pacific Islander, American Indian/Alaska Native, and Other, not specified		

a Continuous Weight Change Variable

© 2022 Davis JS et al. *JAMA Network Open.*

eTable 4. Cox Proportional Hazards Model for Survival by Pre-diagnosis BMI Including Select Tumor Mutations, Where Available

**eTable 4 Cox Proportional Hazards Model for survival by pre-diagnosis BMI in patients with available mutation data**		
N=461	HR (95% CI)	p value
Pre-Diagnosis BMI		
Normal Weight (<25) (Ref)	1.00	
Overweight (25-30)	1.29 (0.98, 1.72)	0.07
Obese (30+)	1.48 (1.07, 2.03)	0.02
Weight Change		
Weight loss (>=10%)	1.09 (0.79, 1.49)	0.61
No Change (Ref)	1.00	
Weight gain (>=10%)	0.74 (0.56, 0.98)	0.04
Age at initial diagnosis		
<45	1.22 (0.90, 1.66)	0.20
45-65 (Ref)	1.00	
65+	1.44 (1.08, 1.92)	0.01
Sex		
Male (Ref)	1.00	
Female	1.04 (0.81, 1.32)	0.78
Race/Ethnicity		
NH African American and Hispanic	0.87 (0.64, 1.18)	0.37
NH Caucasian (Ref)	1.00	
Other	0.76 (0.49, 1.19)	0.23
Stage at initial diagnosis		
I/II (Ref)	1.00	
III	1.24 (0.86, 1.79)	0.24
IV	1.47 (1.06, 2.04)	0.02
KRAS Mutation Status		
WT (Ref)	1.00	
Canonical mutation	1.45 (1.16, 1.82)	0.001
PIK3CA Mutation Status		
WT (Ref)	1.00	
Any Mutation	0.97 (0.75, 1.26)	0.82
NH – Non-Hispanic, Other Race includes Asian/Pacific Islander, American Indian/Alaska Native, and Other, not specified		

© 2022 Davis JS et al. *JAMA Network Open.*

eTable 5. Cox Proportional Hazards Model by Pre-diagnosis Weight Using a 70 kg Cutoff for Comparison With Published Results From Prevention Trials

**eTable 5, Cox proportional Hazards model for survival by pre-diagnosis weight of 70kg**		
N=649	HR (95% CI)	p value
Pre-Diagnosis weight		
<70kg	0.78 (0.61, 1.00)	0.05
70kg+ (Ref)	1.00	
Weight Change		
Weight loss (>=10%)	1.15 (0.89, 1.48)	0.29
No Change (Ref)	1.00	
Weight gain (>=10%)	0.74 (0.59, 0.93)	0.01
Age at initial diagnosis		
<45	1.14 (0.89, 1.47)	0.30
45-65 (Ref)	1.00	
65+	1.38 (1.07, 1.77)	0.01
Sex		
Male (Ref)	1.00	
Female	1.15 (0.91, 1.44)	0.25
Race/Ethnicity		
NH African American and Hispanic	0.95 (0.73, 1.23)	0.70
NH Caucasian (Ref)	1.00	
Other	0.84 (0.57, 1.24)	0.39
Stage at initial diagnosis		
I/II (Ref)	1.00	
III	1.23 (0.90, 1.69)	0.20
IV	1.44 (1.07, 1.93)	0.02
NH – Non-Hispanic, Other Race includes Asian/Pacific Islander, American Indian/Alaska Native, and Other, not specified		

© 2022 Davis JS et al. *JAMA Network Open.*

eTable 6. Cox Proportional Hazards Model by Pre-diagnosis Weight Using a 70 kg Cutoff and Post-diagnosis Aspirin Use, Including an Interaction Term

**eTable 6 Cox Proportional Hazards Model Including interaction term between pre-diagnosis weight 70kg and post-diagnosis aspirin use**			
N=649		HR (95% CI)	p value
Current regular aspirin use*pre-diagnosis 70kg weight			
	No Aspirin Use(ref)	1.00	
	Aspirin Use <70kg pre-diagnosis	0.84 (0.64, 1.10)	
	Aspirin Use ≥70kg pre-diagnosis	0.75 (0.50, 1.12)	
Weight change			
	Weight loss (>= 10%)	1.16 (0.89, 1.50)	0.27
	No Change (Ref)	1.00	
	Weight gain (>=10%)	0.75 (0.60, 0.95)	0.02
Age at initial diagnosis			
	<45	1.12 (0.87, 1.44)	0.39
	45-65 (Ref)	1.00	
	65+	1.39 (1.08, 1.78)	0.01
Sex			
	Male (Ref)	1.00	
	Female	1.11 (0.88, 1.41)	0.38
Race/Ethnicity			
	NH African American or Hispanic	0.94 (0.72, 1.22)	0.63
	NH Caucasian (Ref)	1.00	
	Other	0.81 (0.55, 1.20)	0.30
Stage at initial diagnosis			
	I/II (Ref)	1.00	
	III	1.23 (0.90, 1.69)	0.20
	IV	1.42 (1.06, 1.91)	0.02
NH – Non-Hispanic, Other Race includes Asian/Pacific Islander, American Indian/Alaska Native, and Other, not specified			

© 2022 Davis JS et al. *JAMA Network Open.*

eTable 7. Cox Proportional Hazards Model by Pre-diagnosis BMI, Excluding Underweight (<18 kg/m^2)

**eTable 7 Cox Proportional Hazards Model for Survival by Pre-diagnosis BMI, Excluding Underweight Patients**			
N=642		HR (95% CI)	p value
Pre-Diagnosis BMI			
	Normal Weight (<25) (Ref)	1.00	
	Overweight (25-30)	1.27 (1.00, 1.62)	0.05
	Obese (30+)	1.47 (1.12, 1.93)	0.006
Weight Change			
	Weight loss (>=10%)	1.09 (0.84, 1.42)	0.51
	No Change (Ref)	1.00	
	Weight gain (>=10%)	0.76 (0.60, 0.95)	0.02
Age at initial diagnosis			
	<45	1.16 (0.90, 1.50)	0.26
	45-65 (Ref)	1.00	
	65+	1.36 (1.06, 1.75)	0.02
Sex			
	Male (Ref)	1.00	
	Female	1.10 (0.90, 1.35)	0.36
Race/Ethnicity			
	NH African American and Hispanic	0.91 (0.70, 1.19)	0.50
	NH Caucasian (Ref)	1.00	
	Other	0.81 (0.55, 1.20)	0.30
Stage at initial diagnosis			
	I/II (Ref)	1.00	
	III	1.28 (0.93, 1.77)	0.13
	IV	1.48 (1.10, 2.0)	0.009
NH – Non-Hispanic, Other Race includes Asian/Pacific Islander, American Indian/Alaska Native, and Other, not specified			

© 2022 Davis JS et al. *JAMA Network Open.*

eTable 8. Cox Proportional Hazards Model by Pre-diagnosis BMI and Post-diagnosis Aspirin Use, Excluding Underweight (<18 kg/m^2)

**eTable 8 Cox Proportional Hazards Model Including interaction term between pre-diagnosis BMI and post-diagnosis aspirin use, excluding underweight patients (BMI<18)**			
N=642		HR (95% CI)	p value
Current regular aspirin use*pre-diagnosis BMI weight			
	No Aspirin Use(ref)	1.00	
	Aspirin Use Normal BMI (<25)	0.58 (0.38, 0.90)	
	Aspirin Use Overweight BMI (25-30)	1.05 (0.74, 1.49)	
	Aspirin Use Obese BMI (30+)	0.72 (0.48, 1.08)	
Weight change			
	Weight loss (>= 10%)	1.11 (0.86, 1.45)	0.43
	No Change (Ref)	1.00	
	Weight gain (>=10%)	0.79 (0.63, 1.00)	0.05
Age at initial diagnosis			
	<45	1.13 (0.87, 1.46)	0.36
	45-65 (Ref)	1.00	
	65+	1.38 (1.07, 1.77)	0.01
Sex			
	Male (Ref)	1.00	
	Female	1.07 (0.87, 1.32)	0.52
Race/Ethnicity			
	NH African American or Hispanic	0.89 (0.69, 1.16)	0.41
	NH Caucasian (Ref)	1.00	
	Other	0.78 (0.52, 1.15)	0.21
Stage at initial diagnosis			
	I/II (Ref)	1.00	
	III	1.26 (0.92, 1.74)	0.15
	IV	1.46 (1.08, 1.96)	0.01
NH – Non-Hispanic, Other Race includes Asian/Pacific Islander, American Indian/Alaska Native, and Other, not specified			

© 2022 Davis JS et al. *JAMA Network Open.*

eTable 9. Cox Proportional Hazards Model by Pre-diagnosis BMI and Post-diagnosis Aspirin Use, Reclassifying Users With <81 mg/day as Non-users

**eTable 9 Cox Proportional Hazards Model Including interaction term between pre-diagnosis BMI and post-diagnosis aspirin use, reclassifying very low aspirin use as non-use**			
N=649		HR (95% CI)	p value
Current regular aspirin use*pre-diagnosis BMI weight			
	No Aspirin Use(ref)	1.00	
	Aspirin Use Normal BMI (<25)	0.53 (0.34, 0.84)	
	Aspirin Use Overweight BMI (25-30)	1.02 (0.71, 1.46)	
	Aspirin Use Obese BMI (30+)	0.72 (0.48, 1.08)	
Weight change			
	Weight loss (>= 10%)	1.10 (0.85, 1.44)	0.46
	No Change (Ref)	1.00	
	Weight gain (>=10%)	0.78 (0.62, 0.99)	0.04
Age at initial diagnosis			
	<45	1.15 (0.89, 1.48)	0.28
	45-65 (Ref)	1.00	
	65+	1.36 (1.06, 1.75)	0.02
Sex			
	Male (Ref)	1.00	
	Female	1.07 (0.87, 1.31)	0.52
Race/Ethnicity			
	NH African American or Hispanic	0.89 (0.68, 1.16)	0.38
	NH Caucasian (Ref)	1.00	
	Other	0.79 (0.54, 1.17)	0.19
Stage at initial diagnosis			
	I/II (Ref)	1.00	
	III	1.24 (0.90, 1.71)	0.19
	IV	1.43 (1.06, 1.92)	0.02
NH – Non-Hispanic, Other Race includes Asian/Pacific Islander, American Indian/Alaska Native, and Other, not specified			

© 2022 Davis JS et al. *JAMA Network Open.*

eTable 10. Cox Proportional Hazards Model by Pre-diagnosis BMI, Restricted to Patients Initially Diagnosed With Stage IV Disease

**eTable 10 Cox Proportional Hazards Model for Survival by Pre-diagnosis BMI in Patients Initially Diagnosed with Stage IV Disease**			
N = 384		HR (95% CI)	p value
Pre-Diagnosis BMI			
	Underweight/Normal Weight (<25) (Ref)	1.00	
	Overweight (25-30)	1.20 (0.88, 1.65)	0.25
	Obese (30+)	1.30 (0.89, 1.89)	0.18
Weight Change			
	Weight loss (>=10%)	1.72 (0.82, 1.70)	0.39
	No Change (Ref)	1.00	
	Weight gain (>=10%)	0.69 (0.51, 0.94)	0.02
Age at initial diagnosis			
	<45	1.14 (0.83, 1.56)	0.41
	45-65 (Ref)	1.00	
	65+	1.06 (0.75, 1.49)	0.74
Sex			
	Male (Ref)	1.00	
	Female	1.01 (0.77, 1.31)	0.97
Race/Ethnicity			
	NH African American and Hispanic	0.99 (0.70, 1.40)	0.95
	NH Caucasian (Ref)	1.00	
	Other	0.73 (0.44, 1.22)	0.23
NH – Non-Hispanic, Other Race includes Asian/Pacific Islander, American Indian/Alaska Native, and Other, not specified			

© 2022 Davis JS et al. *JAMA Network Open.*

eTable 11. Cox Proportional Hazards Model by Pre-diagnosis BMI and Post-diagnosis Aspirin Use, Restricted to Patients Initially Diagnosed With Stage IV Disease

**eTable 11 Cox Proportional Hazards Model Including interaction term between pre-diagnosis BMI and post-diagnosis aspirin use, in patients initially diagnosed with stage IV disease**			
N=384		HR (95% CI)	p value
Current regular aspirin use*pre-diagnosis BMI weight			
	No Aspirin Use(ref)	1.00	
	Aspirin Use Normal BMI (<25)	0.30 (0.13, 0.66)	
	Aspirin Use Overweight BMI (25-30)	1.11 (0.70, 1.74)	
	Aspirin Use Obese BMI (30+)	0.81 (0.46, 1.43)	
Weight change			
	Weight loss (>= 10%)	1.21 (0.84, 1.75)	0.31
	No Change (Ref)	1.00	
	Weight gain (>=10%)	0.78 (0.57, 1.07)	0.12
Age at initial diagnosis			
	<45	1.11 (0.81, 1.52)	0.52
	45-65 (Ref)	1.00	
	65+	1.09 (0.77, 1.53)	0.64
Sex			
	Male (Ref)	1.00	
	Female	0.96 (0.73, 1.25)	0.76
Race/Ethnicity			
	NH African American or Hispanic	0.97 (0.69, 1.37)	0.85
	NH Caucasian (Ref)	1.00	
	Other	0.67 (0.40, 1.13)	0.14
NH – Non-Hispanic, Other Race includes Asian/Pacific Islander, American Indian/Alaska Native, and Other, not specified			

© 2022 Davis JS et al. *JAMA Network Open.*
